# Comment on: Maternal Exposure to Domestic Hair Cosmetics and Occupational Endocrine Disruptors Is Associated with a Higher Risk of Hypospadias in the Offspring. *Int. J. Environ. Res. Public Health* 2017, *14*, 27

**DOI:** 10.3390/ijerph14091061

**Published:** 2017-09-14

**Authors:** Mark Elwood, Dillon O’Brien, Katie-Anne Budgen

**Affiliations:** School of Population Health, University of Auckland, Auckland 1142, New Zealand; dillonobrien@outlook.co.nz (D.O.); ktbudgen@gmail.com (K.-A.B.)

There are considerable inconsistencies in the results of Haraux et al. [1]. For one of the main exposures of interest, maternal exposure to hair cosmetics, a univariate odds ratio (OR) of 1.8 is given in the text and in Table 4; yet the data shown give a univariate OR of 0.74 (Wolff 95% confidence limits 0.37 to 1.48). Similarly, the OR for colouring shampoo is given as 1.3, yet the data give 1.61 (limits 0.74 to 3.48). It is also notable that for cases, all mothers reported as using hair cosmetics (16) used hairspray, with most (13) also using colouring shampoo; while for controls, only 37 of 51 users of hair cosmetics used hairspray. There is a considerable difference between the amount of missing data on use of hair cosmetics for cases (10%, 6/57 missing) and for controls (17%, 28 missing). For the other exposure of main interest, occupational exposure to endocrine disrupting chemicals (EDCs), 11% of cases compared to 4% of controls had missing data on whether they were working during the pregnancy, but fewer had missing data on the specific question of EDCs; so some mothers with no information on occupation appear to be classified in terms of occupational EDC exposure.

Another unusual feature is that the univariate odds ratio of 1.8, (or 0.74), for hair cosmetics changes to an OR of 6.11 on multivariate analysis. For occupational exposure to EDCs, the univariate OR of 3.1 changes to 9.64 in the multivariate analysis. Such a large change is unusual and shows severe negative confounding; it would be interesting to know the responsible confounders. In a similar study of hypospadias and occupational exposure to hair sprays, the univariate and adjusted ORs were similar, 2.30 and 2.39, with both studies adjusting for birth weight and folate intake [2].

The participation rates in this study are undocumented, and the results depend on questionnaires completed by the parents, presumably in the hospital environment, shortly after birth, when anxiety about the child would be high and the potential for differential responses considerable.
